# Supplementary material for: Molecular architecture of glideosome and nuclear F-actin in Plasmodium falciparum
Source: EMBO Rep. 2025 Mar 24;26(8):1984–96. doi: 10.1038/s44319-025-00415-7 (PMC12019134; doi:10.1038/s44319-025-00415-7)
Supplement: Supplementary file 2 — Movie EV1 [file 44319_2025_415_MOESM2_ESM.zip › Movie EV1 legend.docx]

**Movie EV1:** Movie showing a tomogram and a surface representation of *Plasmodium falciparum* sporozoite apical pole generated by manual segmentation and subvolume averaging. There are 2 actin filaments visible in the oblique section through the volume shown towards the end of the movie, both apparently nucleated by preconoidal rings. One was not analysed by subvolume averaging due to its short length. Scale bar represents 100 nm.
