# Supplementary material for: Molecular architecture of glideosome and nuclear F-actin in Plasmodium falciparum
Source: EMBO Rep. 2025 Mar 24;26(8):1984–96. doi: 10.1038/s44319-025-00415-7 (PMC12019134; doi:10.1038/s44319-025-00415-7)
Supplement: Supplementary file 3 — Movie EV2 [file 44319_2025_415_MOESM3_ESM.zip › Movie EV2 legend.docx]

**Movie EV2:** Movie moving through a tomogram showing two *Plasmodium falciparum* sporozoite basal poles. Basal pores are seen with an example of an actin filament sitting within one pore.
