# Supplementary material for: Molecular architecture of glideosome and nuclear F-actin in Plasmodium falciparum
Source: EMBO Rep. 2025 Mar 24;26(8):1984–96. doi: 10.1038/s44319-025-00415-7 (PMC12019134; doi:10.1038/s44319-025-00415-7)
Supplement: Supplementary file 4 — Movie EV3 [file 44319_2025_415_MOESM4_ESM.zip › Movie EV3 legend.docx]

**Movie EV3:** The same volume as shown in Video 2, this time also displayed with surface representation of segmented membranes and backplotted actin filaments. Scale bar represents 100 nm.
