# Supplementary material for: Molecular architecture of glideosome and nuclear F-actin in Plasmodium falciparum
Source: EMBO Rep. 2025 Mar 24;26(8):1984–96. doi: 10.1038/s44319-025-00415-7 (PMC12019134; doi:10.1038/s44319-025-00415-7)
Supplement: Supplementary file 6 — Movie EV5 [file 44319_2025_415_MOESM6_ESM.zip › Movie EV5 legend.docx]

**Movie EV5:** The same volume as in Video 4, rotated to move through all regions of the pellicle and focusing on an area with 2 putative myosins bound to an actin filament and a thin pellicular filament. The second part of this video shows this volume overlayed with a backplotted average of an actin filament and a segmented PfMyoA motor domain (EMD-10590) for size comparison.
