# Supplementary figures and images for: Molecular architecture of glideosome and nuclear F-actin in Plasmodium falciparum
Source: EMBO Rep. 2025 Mar 24;26(8):1984–96. doi: 10.1038/s44319-025-00415-7 (PMC12019134; doi:10.1038/s44319-025-00415-7)

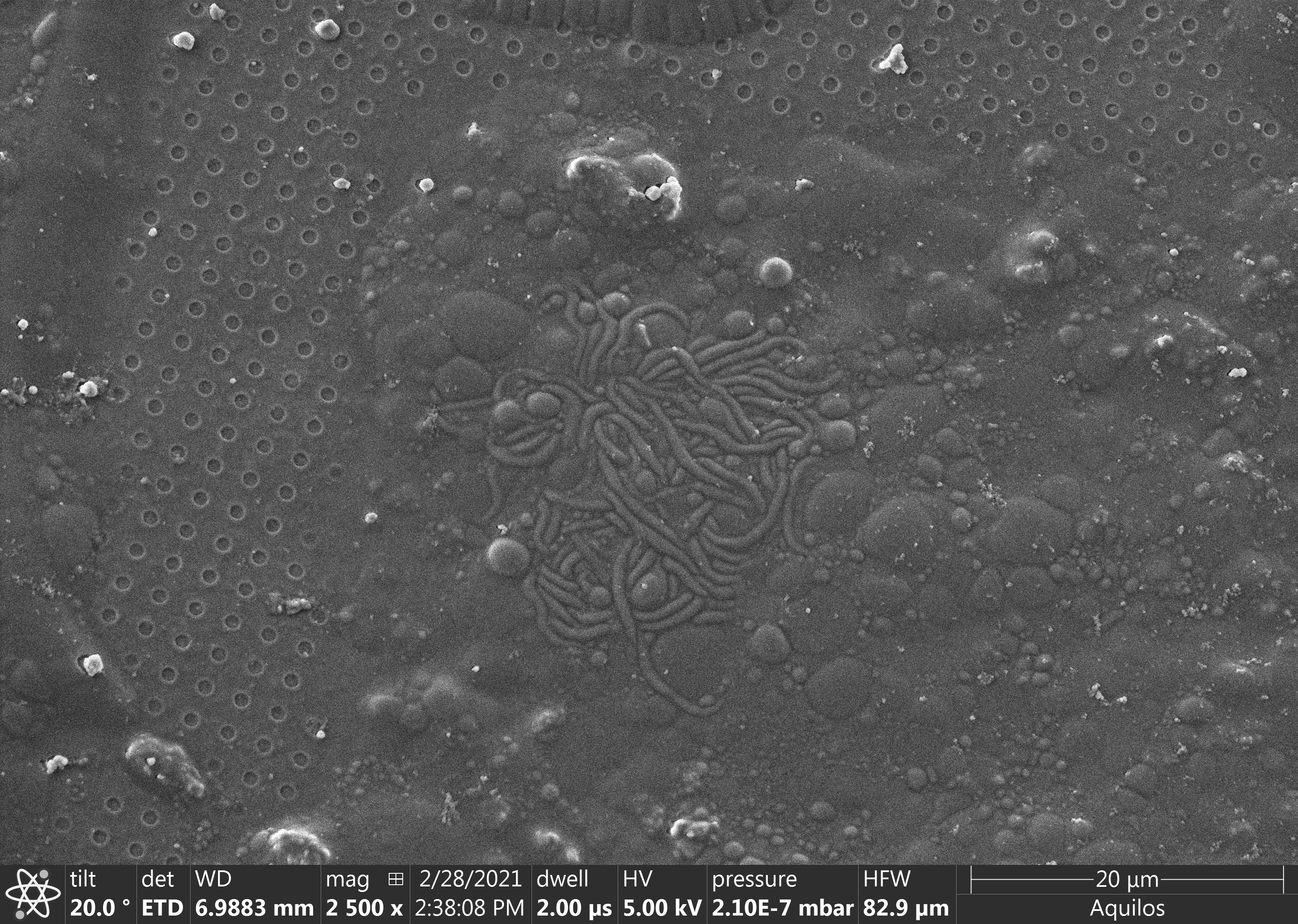

Supplement: Supplementary file 12 — Figure EV1 Source Data [file 44319_2025_415_MOESM12_ESM.zip › figure EV1/sporozoites_SEM_015.tif]

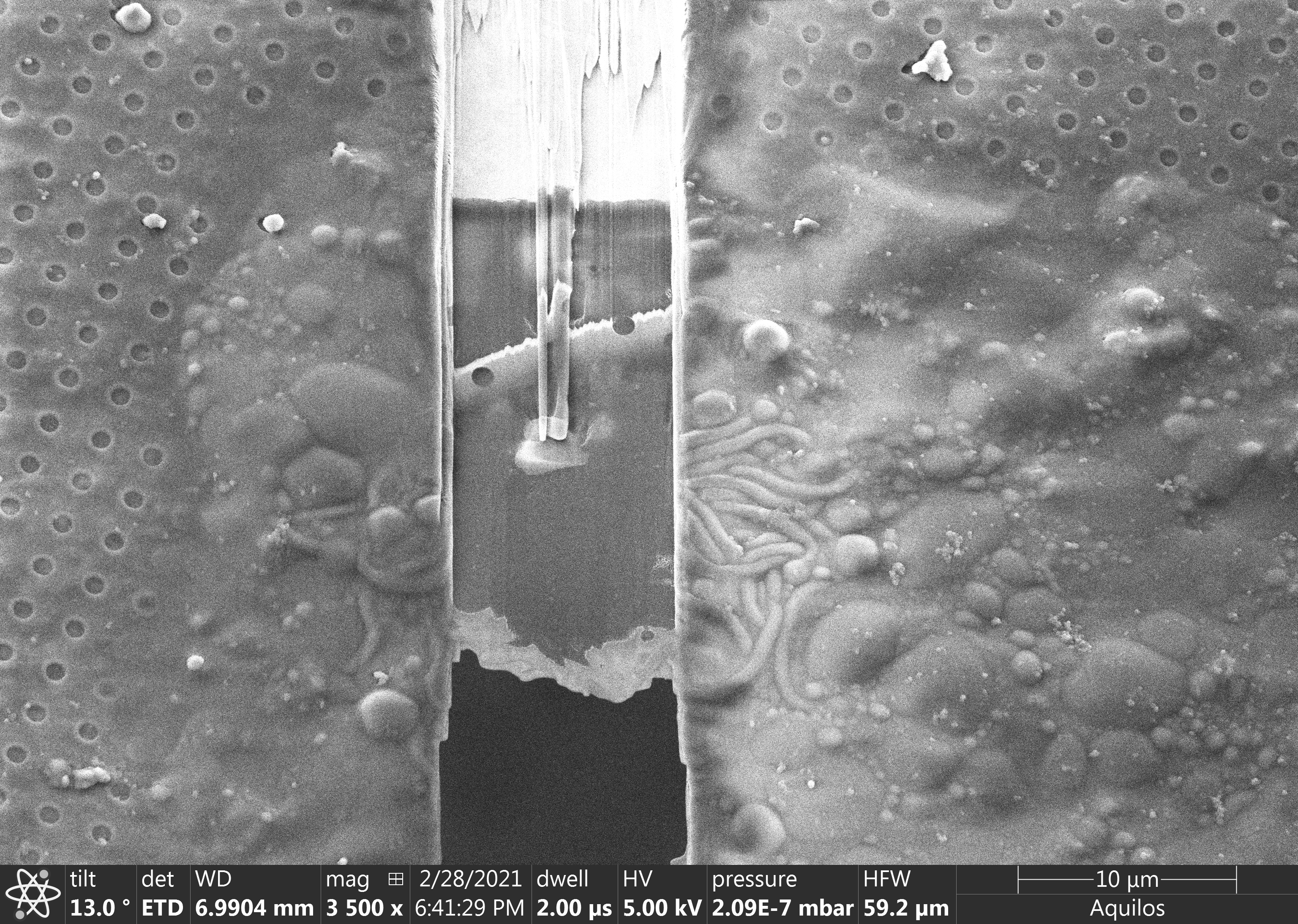

Supplement: Supplementary file 12 — Figure EV1 Source Data [file 44319_2025_415_MOESM12_ESM.zip › figure EV1/sporozoites_SEM_047.tif]

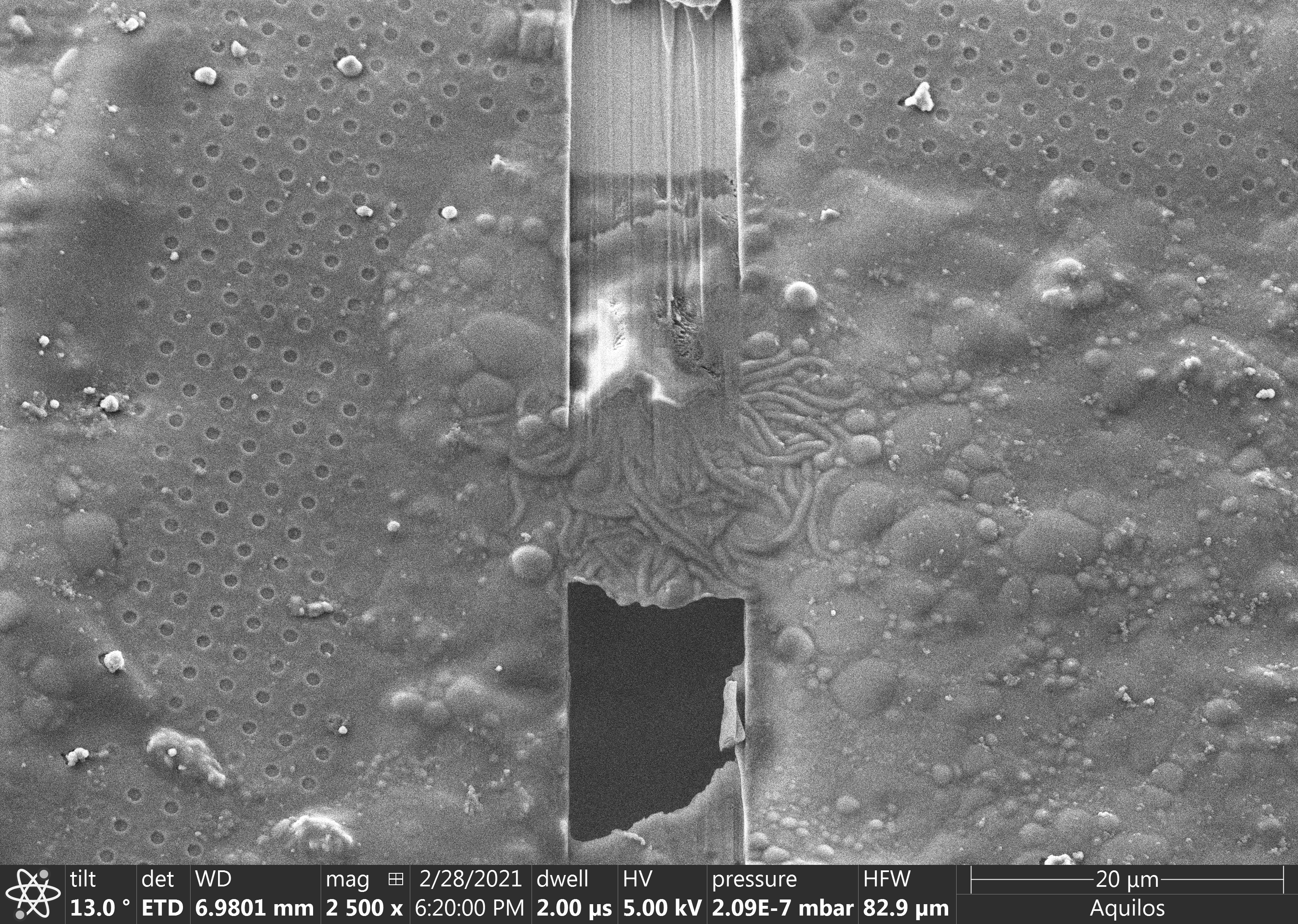

Supplement: Supplementary file 12 — Figure EV1 Source Data [file 44319_2025_415_MOESM12_ESM.zip › figure EV1/sporozoites_SEM_044.tif]
