# Supplementary material for: Molecular architecture of glideosome and nuclear F-actin in Plasmodium falciparum
Source: EMBO Rep. 2025 Mar 24;26(8):1984–96. doi: 10.1038/s44319-025-00415-7 (PMC12019134; doi:10.1038/s44319-025-00415-7)
Supplement: Supplementary file 16 — Expanded View Figures [file 44319_2025_415_MOESM16_ESM.pdf]

Expanded View Figures

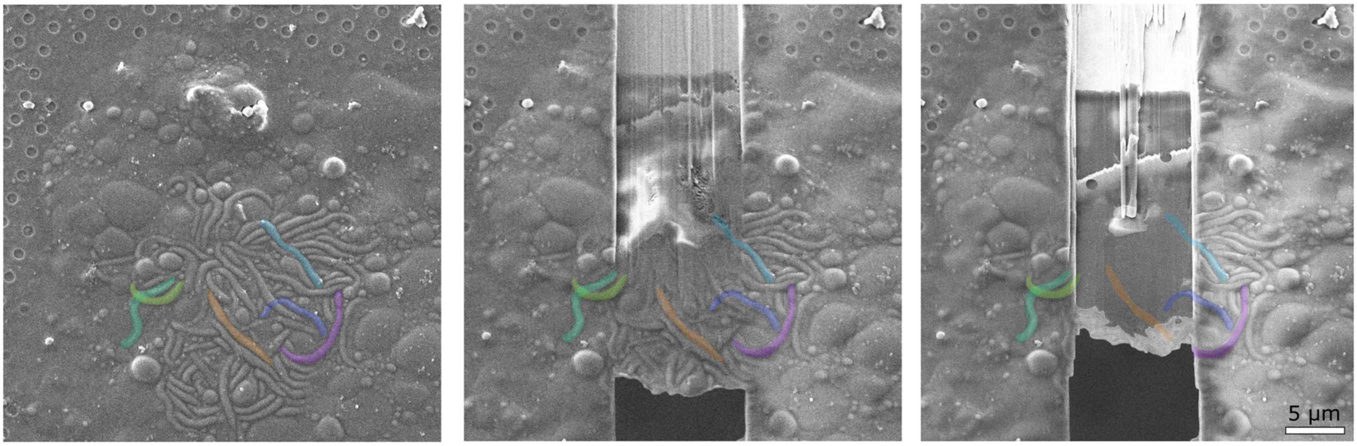

Figure EV1. FIB-milling sporozoites.

Left: A pile of sporozoites in the SEM. A few individual sporozoites are coloured consistently in all three images. Middle: SEM image during the FIB-milling process. Right: SEM image of the final polished lamella. Source data are available online for this figure.

 © The Author(s)

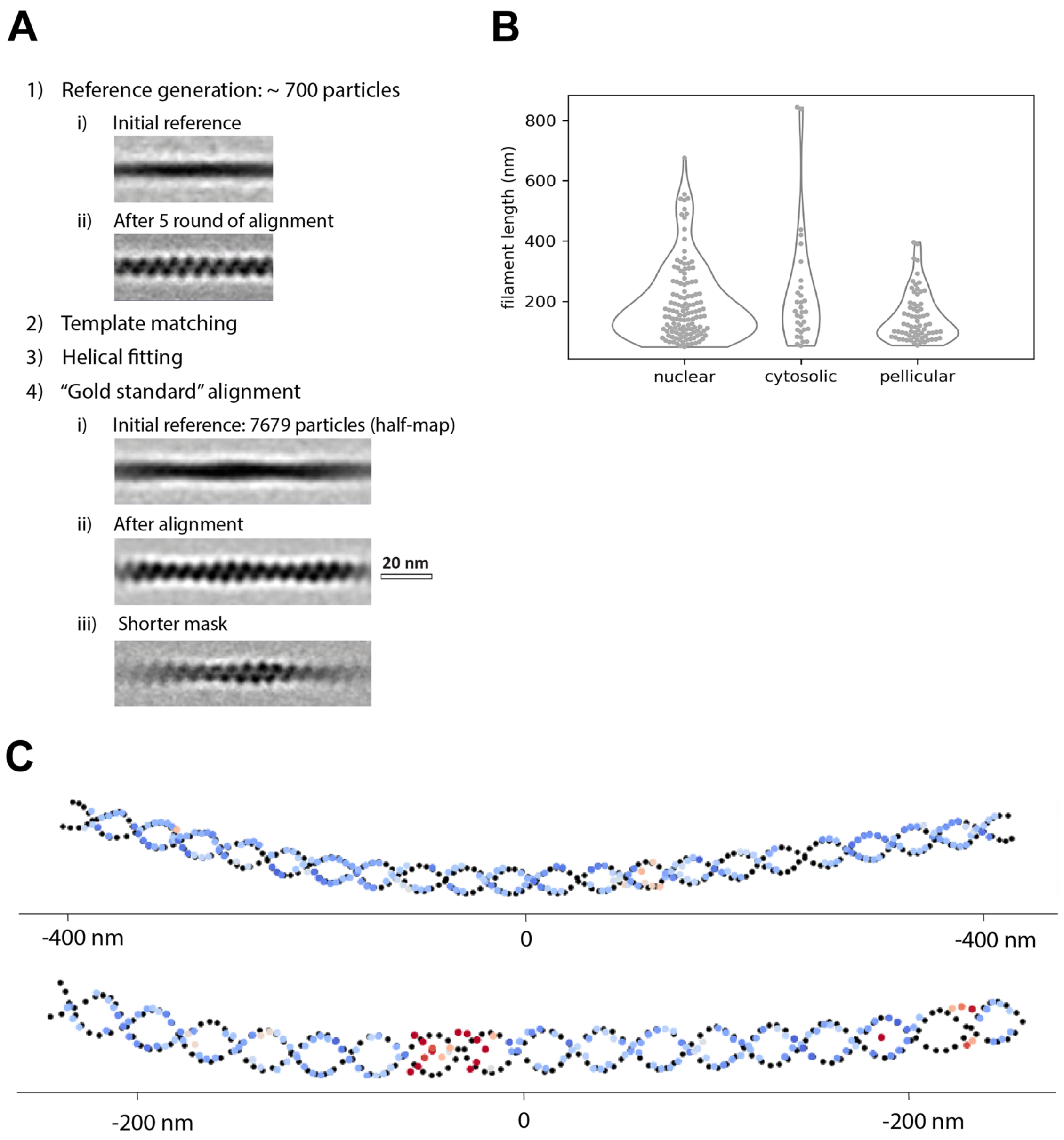

Figure EV2. Subvolume averaging of actin.

(**A**) A rough outline of the workflow used for subvolume averaging of actin, with some reference volumes shown. (**B**) Size distribution of F-actin in indicated subcellular locations derived from subvolume averaging coordinates. Note that especially longer filaments were frequently truncated during FIB-milling causing the distribution to be skewed towards shorter sizes. Two-sided t-test *p*-values are 0.4 and 1×10^{-4} comparing nuclear and cytosolic, and nuclear and pellicular, respectively. Caution should be used interpreting the significance of the difference due to some filaments being truncated. (**C**) Diagnostic plots of helical fitting of two longer filaments. Black dots show best fit positions of actin subunits. Overlaid are positions measured by subvolume averaging coloured from blue to red based on distance to the nearest model point. Source data are available online for this figure.

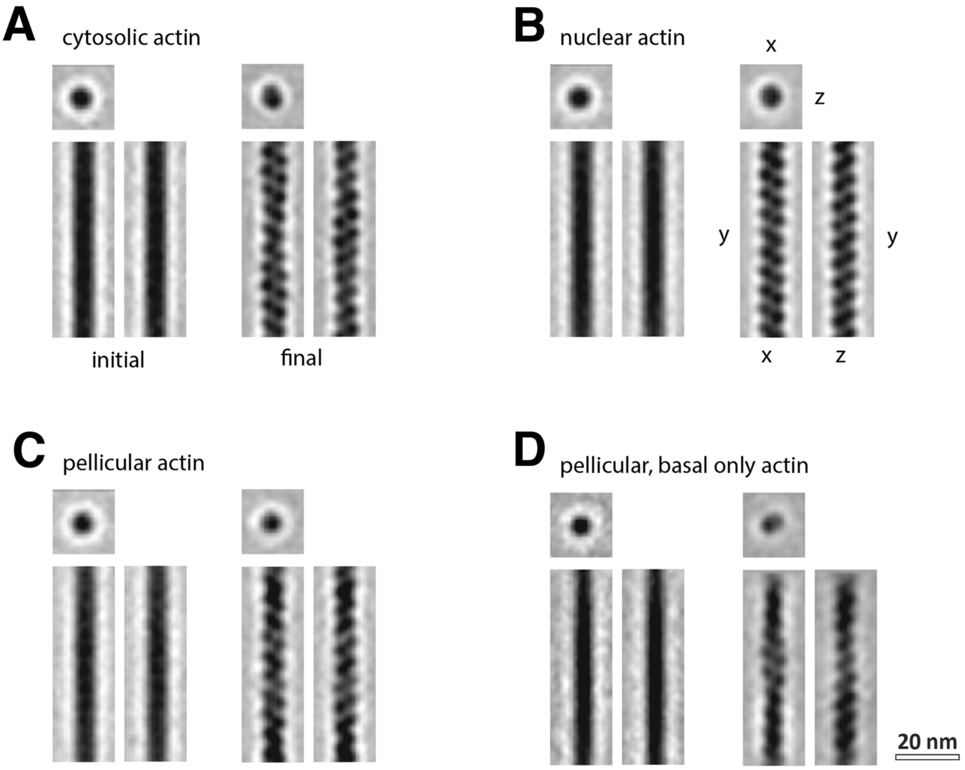

Figure EV3. Subvolume averages of actin from different subcellular compartments.

Actin filaments used for subvolume averaging (Figs. 1E and EV2) were manually split into classes based on their subcellular localization: (**A**) cytosol, (**B**) nucleus, (**C**) pellicular space, (**D**) pellicular space at the basal end. The particle Y axis (long filament axis) orientations were randomised and independent references were generated for each class (left hand side). Alignment was performed without refining orientations. Source data are available online for this figure.

 © The Author(s)

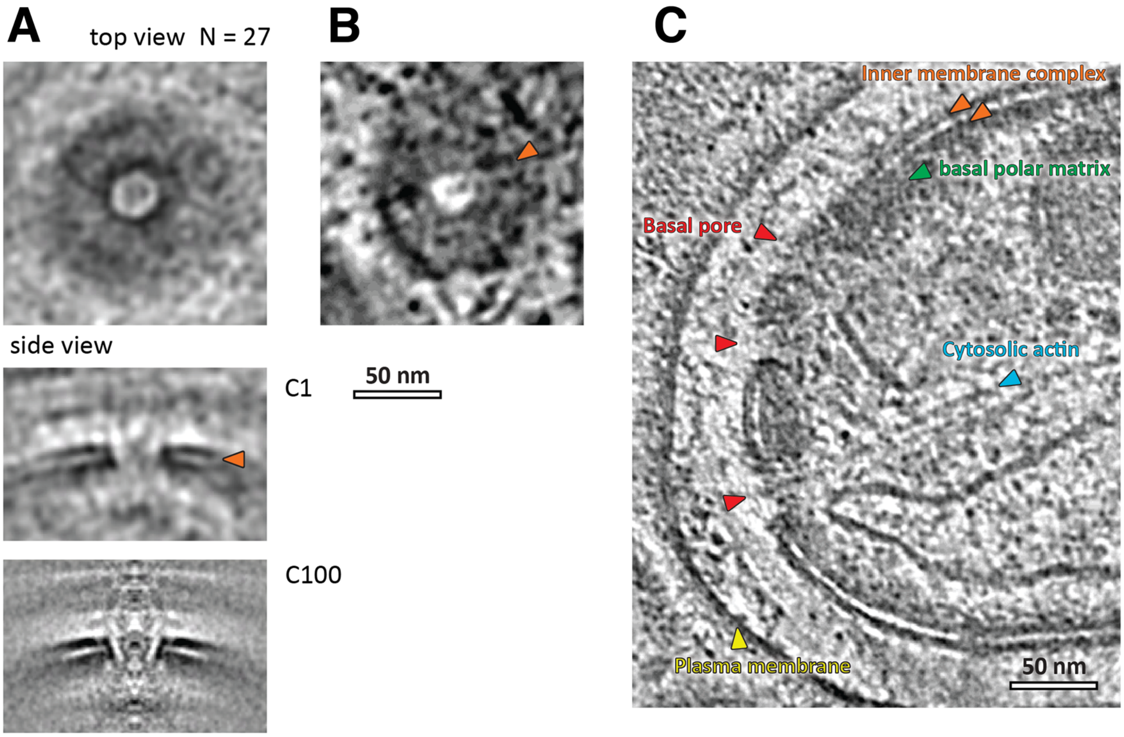

Figure EV4. Basal pores form 25 nm diameter connections between the cytoplasm and pellicular space.

(**A**) Subvolume average of 27 particles. No symmetry is evident at this resolution. (**B**) A tangential slice through a single pore. (**C**) Slice through a basal end of a sporozoite (also in Fig. 2B) showing three basal pores. Source data are available online for this figure.
